# Labeling Adipose-Derived Stem Cells with Hoechst 33342: Usability and Effects on Differentiation Potential and DNA Damage

**DOI:** 10.1155/2016/6549347

**Published:** 2016-06-08

**Authors:** P. Schendzielorz, K. Froelich, K. Rak, T. Gehrke, A. Scherzad, R. Hagen, A. Radeloff

**Affiliations:** Comprehensive Hearing Center, Department of Otorhinolaryngology, Plastic, Aesthetic and Reconstructive Head and Neck Surgery, University of Würzburg, Josef-Schneider-Strasse 11, 97080 Würzburg, Germany

## Abstract

Adipose-derived stem cells (ASCs) have been extensively studied in the field of stem cell research and possess numerous clinical applications. Cell labeling is an essential component of various experimental protocols and Hoechst 33342 (H33342) represents a cost-effective and easy methodology for live staining. The purpose of this study was to evaluate the labeling of rat ASCs with two different concentrations of H33342 (0.5 *μ*g/mL and 5 *μ*g/mL), with particular regard to usability, interference with cell properties, and potential DNA damage. Hoechst 33342 used at a low concentration of 0.5 *μ*g/mL did not significantly affect cell proliferation, viability, or differentiation potential of the ASCs, nor did it cause any significant DNA damage as measured by the olive tail moment. High concentrations of 5 *μ*g/mL H33342, however, impaired the proliferation and viability of the ASCs, and considerable DNA damage was observed. Undesirable colabeling of unlabeled cocultivated cells was seen in particular with higher concentrations of H33342, independent of varying washing procedures. Hence, H33342 labeling with lower concentrations represents a usable method, which does not affect the tested cell properties. However, the colabeling of adjacent cells is a drawback of the technique.

## 1. Introduction

Adipose-derived stem cells (ASCs) have been focused on in various fields of stem cell research and clinical applications. These cells can be harvested from adult individuals with low donor-site morbidity and thus can be transplanted autologously. ASCs are multipotent and are able to undergo multilineage differentiation, for example, into osteogenic, chondrogenic, and adipogenic directions. This differentiation potential, the adherence to plastic surfaces, and the expression of certain CD-surface markers are required characteristics for defining ASCs [1]. For numerous research purposes, the identification of transplanted ASCs is essential. Ideally, the labeling process meets the following criteria: (1) it should not interfere with the proliferation and vitality of labeled cells, (2) it should not impair the differentiation potential, (3) it should be persistent long-term, and (4) it should not contaminate adjacent cells or structures. Fluorescent dyes are commonly used for cell labeling, and, among others, fluorochromes like PKH26, CSFE [2], or Dil [3] were investigated for labeling ASCs. Another material that is often used as a cell tracker is Hoechst 33342 (H33342), which has been widely used in lymphocyte migration assays [4, 5]. Hoechst 33342 belongs to the category of bisbenzimides, penetrates membranes, and binds to Adenine-Thymine- (AT-) rich regions of the DNA. The material exhibits high staining persistence and fluorescence and has been used for in vivo experiments and in fixed tissue [5]. Its main advantage is that it labels the cell nucleus and thus allows for quantification of labeled cells. The purpose of this study is to investigate the labeling of ASCs with two different concentrations of H33342, with particular regard to potential interference with cell viability, proliferation, and differentiation potential.

## 2. Materials and Methods

### 2.1. Isolation and Culture of Rat ASC

The procedure was performed in accordance with the German Protection of Animals Act and reported to the responsible authorities (Regierung von Unterfranken, Wuerzburg, Germany). ASCs were isolated from adipose tissue of the neck of 5 Sprague Dawley rats (Charles River, Sulzfeld, Germany). After removal of the hackles in the dorsal neck region, a horizontal skin incision was performed. Adipose tissue was harvested and transferred into sterile phosphate buffered saline (PBS, Roche, Mannheim, Germany) with 2% penicillin/streptomycin (Biochrom AG, Berlin, Germany) until processed. The isolation procedure was performed as previously described [6] with slight modifications. Briefly, the adipose tissue was finely minced and washed with PBS. After digestion with 0.1 mg/mL of collagenase P (Roche Diagnostics GmbH, Mannheim, Germany) for 3 hours at 37°C, the adipose cell fraction was removed by centrifugation at 340 times gravity (×g). Erythrocytes were lysed by erythrocyte lysis buffer (Merck, Darmstadt, Germany). The remaining cells were washed with PBS and resuspended in ASC medium consisting of Dulbecco's Modified Eagle's Medium (DMEM; Gibco Invitrogen, Karlsruhe, Germany), 10% fetal calf serum (FCS; Linaris, Wertheim-Bettingen, Germany), and 1% penicillin/streptomycin. The cell suspension was filtered with a 100 *μ*m cell strainer (BD Bioscience, Bedford, MA, USA) before plating the cells in culture flasks. After 24 hours, ASCs were rinsed with PBS to remove nonadherent cells and debris. The culture medium was changed every other day. At 80% confluence, cells were detached using 0.25% trypsin solution containing 1 mM EDTA (Gibco Invitrogen) and transferred to the next passage. ASCs from passage 2 were used throughout this study. The experiments were performed with cells of *n* = 5 individuals except as noted otherwise.

### 2.2. Flow Cytometry

Flow cytometry was performed to verify the expression of ASC-specific cell surface markers using the Becton Dickinson FACS-Canto*™* (BD Bioscience, Bedford, MA, USA). For this purpose, 2 × 10^6^ ASCs were incubated for each experiment with antibodies against cluster of differentiation (CD) surface proteins according to the manufacturer's protocol.

Antibodies against CD44 (conjugated with fluorescein (FITC), number 550974, BD Bioscience), CD73 (conjugated with phycoerythrin (PE), number 550257, BD Bioscience), and CD90 (conjugated with PE, number 554101, BD Bioscience) were used as positive markers. Antibodies against CD34 (conjugated with PE, number 550761, BD Bioscience) and CD45 (conjugated with FITC, number 561867, BD Bioscience) were used as negative markers.

### 2.3. Hoechst 33342 Labeling

ASCs were labeled with H33342 (Sigma-Aldrich, Steinheim, Germany) at 2 different concentrations (0.5 *μ*g/mL and 5 *μ*g/mL). To accomplish this, attached ASCs were incubated with H33342 solution of the respective concentration for 90 minutes at 37°C in an incubator. After staining, the cells were rinsed twice with PBS and ASC medium was added.

### 2.4. Proliferation Assay

For determining the proliferation properties, ASCs (unlabeled or labeled with 0.5 *μ*g/mL or 5 *μ*g/mL H33342) were cultivated in culture flasks with ASC medium at 37°C with 5% CO_2_ in an incubator. Medium was changed every other day. After 3, 7, 14, 21, and 28 days, labeled and unlabeled cells were detached with trypsin solution and counted using an automated cell counter (Casey Technologies, Innovatis AG, Reutlingen, Germany). Then, 5 × 10^5^ cells of each group were transferred to a new culture flask.

### 2.5. Cell Viability Stainings

The cell viability was evaluated with 2 different techniques. First, the fluorescein diacetate/propidium iodide (FDA/PI, Sigma-Aldrich) method was performed at days 1, 3, 7, 14, and 28 that allows for identifying living and dead cells. For this, a working solution was prepared containing 0.1 mg/mL FDA and 0.01 mg/mL PI in PBS. Cells were detached with trypsin and counted, and 1 × 10^4^ cells were resuspended in 60 *μ*L DMEM in opaque microtubes (Hartenstein, Wuerzburg, Germany). Then, 20 *μ*L of the FDA/PI solution was added to the cell suspension. Samples were incubated for 30 seconds. Thereafter, 20 *μ*L was transferred onto a microscope slide (Langenbrinck, Emmendingen, Germany) and evaluated with a fluorescence microscope (DMI 4000B, Leica, Wetzlar, Germany). 200 cells were evaluated for each animal.

Second, a 1-(4,5-dimethylthiazol-2-yl)-3,5-diphenylformazan tetrazolium bromide assay (MTT assay, Sigma-Aldrich) was performed at days 3, 7, 14, and 28. This technique provides an indirect measure for the number of proliferating cells. Here, 10^4^ cells/well were plated in 24-well plates (BD Falcon, Heidelberg, Germany) in ASC medium. After 24 hours, the culture medium was aspirated and the cells were rinsed twice with PBS (Roche). The MTT solution was prepared by adding 8 mg of MTT to 10 mL fresh DMEM and 500 *μ*L of MTT solution was added to each well. Four hours later, the MTT solution was removed and each well was incubated with 500 *μ*L isopropanol for 30 min. The extinction values of the isopropanol-diluted formazan were measured at 570 nm wavelength. The viability index was determined by calculating the ratio of the extinction values of labeled cells compared to unlabeled control cells.

### 2.6. Comet Assay

At 24 hours and after 4 weeks, the comet assay was conducted for labeled and control ASCs as described previously [7] to detect DNA strand breaks, alkali labile sites, and incomplete excision repair sites in single cells. Briefly, 10^5^ ASCs were cultivated in 6 wells for each condition and donor. Cells were detached with trypsin solution. After preparing the agarose slides, alkaline cell lysis occurred for 1 h. The slides were placed in the electrophoresis chamber and DNA unwinding occurred. Then, electrophoresis was performed for 20 min. Cells were stained with ethidium bromide (Sigma-Aldrich) after a neutralization phase at pH 7.5. For evaluation of the slides, a fluorescence microscope (Laborlux S, Leica Microsystems, Wetzlar, Germany) with a filter system containing a green excitation filter (515–560 nm), a dichromatic beam splitter (580 nm), and an emission filter (590 nm) at 400-fold magnification were used. To analyze DNA fragmentation, the comet 5.5 image system (Kinetic Imaging, Liverpool, UK) was used. Tail DNA, tail length (TL), and olive tail moment (OTM), which is the product of the median migration distance and the percentage of DNA in the tail, were determined to quantify the DNA fragmentation. Statistical evaluation was based on the OTM values [8].

### 2.7. Multilineage Differentiation Potential

The multilineage differentiation potential was evaluated 24 hours and 2 weeks after cell labeling (*n* = 3). To measure this, adipogenic, osteogenic, and chondrogenic differentiation potential were induced as previously described [3].

Briefly, for adipogenic differentiation, ASCs were seeded in 4-well plates (Greiner Bio-One GmbH, Frickenhausen, Germany) at a density of 2 × 10^4^ cells/cm^2^. The ASC medium was supplemented with 1 *μ*g/mL insulin (PAA, GE Health Life Science, Freiburg, Germany), 10 *μ*M dexamethasone (Sigma-Aldrich), 100 *μ*M indomethacin (Sigma-Aldrich), and 500 *μ*M 1-methyl-3-isobutylxanthine (Sigma-Aldrich) and was replaced every other day. After 2 weeks, staining with oil red O (Sigma-Aldrich/Merck) was performed to identify intracellular lipid vacuoles after adipogenic differentiation [9].

For osteogenic differentiation, ASCs were seeded in 4-well plates at a density of 2 × 10^4^ cells/cm^2^ and differentiation was induced by adding 100 nM dexamethasone, 10 mM *β*-glycerophosphate (Sigma-Aldrich), and 50 *μ*g/mL ascorbic acid-2-phosphate (Sigma-Aldrich) to the ASC medium. Cells were cultured for 3 weeks and medium was replaced every other day. Osteogenic differentiation and extracellular mineral deposition were histologically confirmed by the presence of black nodules after von Kossa staining (Sigma-Aldrich/Merck) and red-stained extracellular calcium deposits after alizarin red (Merck) staining [9].

Chondrogenic differentiation was performed as a 3-dimensional pellet culture. For this, 3 × 10^5^ ASCs in ASC medium were transferred into 15 mL polypropylene tubes (Cellstar, Greiner Bio-One) and centrifuged at 350 g for 8 min to form a cell pellet. Differentiation was induced using a defined chondrogenic differentiation medium consisting of DMEM supplemented with 1% P/S, 100 nM dexamethasone, 100 *μ*g/mL sodium pyruvate (Sigma-Aldrich), 50 *μ*g/mL ascorbic acid-2-phosphate, 40 *μ*g/mL proline (Sigma-Aldrich), ITS-plus (Sigma-Aldrich), and 10 ng/mL TGF-*β*3 (LONZA, Basel, Switzerland). Pellets were then cultured for 3 weeks. After 3 weeks, pellets were fixed with 4% paraformaldehyde (4% PFA, Serva Feinbiochemica, Heidelberg, Germany), washed, and embedded in Tissue-Tek OCT® (Sakura, Alphen aan den Rijn, Netherlands) for cryostat sectioning. Chondrogenic differentiation was evaluated after alcian blue (Sigma-Aldrich) staining of extracellular glycosaminoglycans [9].

As negative controls for all differentiation procedures, ASCs were maintained in ASC medium in monolayer cultures and as pellet cultures. All cultures mentioned above were performed in an incubator at 37°C in a humidified atmosphere containing 5% CO_2_.

### 2.8. Evaluation of Labeling Stability and Contamination

In order to study the labeling stability, 1 × 10^4^ cells labeled with 0.5 *μ*g/mL or 5 *μ*g/mL H33342 were plated in 6-wells with 2 mL ASC medium. The medium was changed every other day. After 3, 14, 28, and 56 days, the cells were rinsed twice with PBS and fixed with 4% PFA. The cells were stained with PI solution as described above. Then, 5 pictures were taken of each well at predefined positions (0°, 90°, 180°, and 270° and centrally) with a fluorescence microscope (DMI 4000B, Leica, Wetzlar, Germany) at 200-fold magnification. Only PI stained cells were counted and the percentage of H33342 labeled cells was evaluated.

H33342 labeled ASCs were cocultivated with unlabeled ASCs to analyze a potential contamination of the formerly unlabeled cells. The experiments were performed with cells from 5 individuals. For this, two experiments were conducted with labeled and unlabeled ASCs (1) in direct proximity on cover slips and (2) separated in different chambers of a transwell system. Both experiments were conducted with varying washing procedures of the labeled ASCs.

For procedure (1), 10 *μ*L of medium containing 1 × 10^4^ labeled ASCs was placed on the left margin and 10 *μ*L of medium containing 1 × 10^4^ unlabeled ASCs was placed on the right margin of each coverslip. Cover slips were placed in 4-well dishes. After 3 hours, 80 *μ*L of ASC medium was added. Before plating, labeled cells were washed in three different ways: (a) labeled cells were rinsed once with PBS and centrifuged, (b) labeled cells were rinsed three times with PBS and centrifuged, and (c) labeled cells were rinsed once with PBS, centrifuged, and cultivated for two days; then, cells were rinsed twice with PBS and centrifuged again.

After 2, 4, and 8 days, cells were fixed with PFA 4% and rinsed twice with PBS. Pictures were taken from both the left and right edge of each cover slip with the fluorescence microscope. The following setting of the camera was retained for all pictures: gain 2.3x; gamma 1.0; magnification 200-fold. The initial exposure time of 100 ms was increased by 100 ms increments until the “primarily unlabeled” cells were well distinguishable.

For procedure (2), 1 × 10^4^ unlabeled cells were plated in 24-well plates of the transwell system and 1 mL ASC medium was added. Then, 1 × 10^4^ labeled ASCs were plated into the inserts. Before plating, the labeled cells were treated as follows: (a) labeled cells were rinsed two times with PBS and centrifuged and (b) labeled cells were rinsed with PBS twice, centrifuged, and cultivated for two days; then, cells were rinsed twice with PBS and centrifuged again.

After 2 and 8 days, the inserts were removed. The primarily unlabeled cells in the wells were fixed with PFA 4% and rinsed twice with PBS. Pictures were taken with the fluorescence microscope with a fixed setting as specified above. The initial exposition time of 100 ms was increased by 100 ms increments up to 500 ms. At each step, it was documented whether labeled cells were detectable. The experiments were conducted in triplicate.

### 2.9. Statistical Evaluation

For statistical evaluation, the Kruskal-Wallis test and a consecutive Dunn's post hoc test were performed. A* p* value of less than 0.05 was considered statistically significant.

## 3. Results

### 3.1. Cell Characterization, Viability, and Proliferation

**Figure 1:**
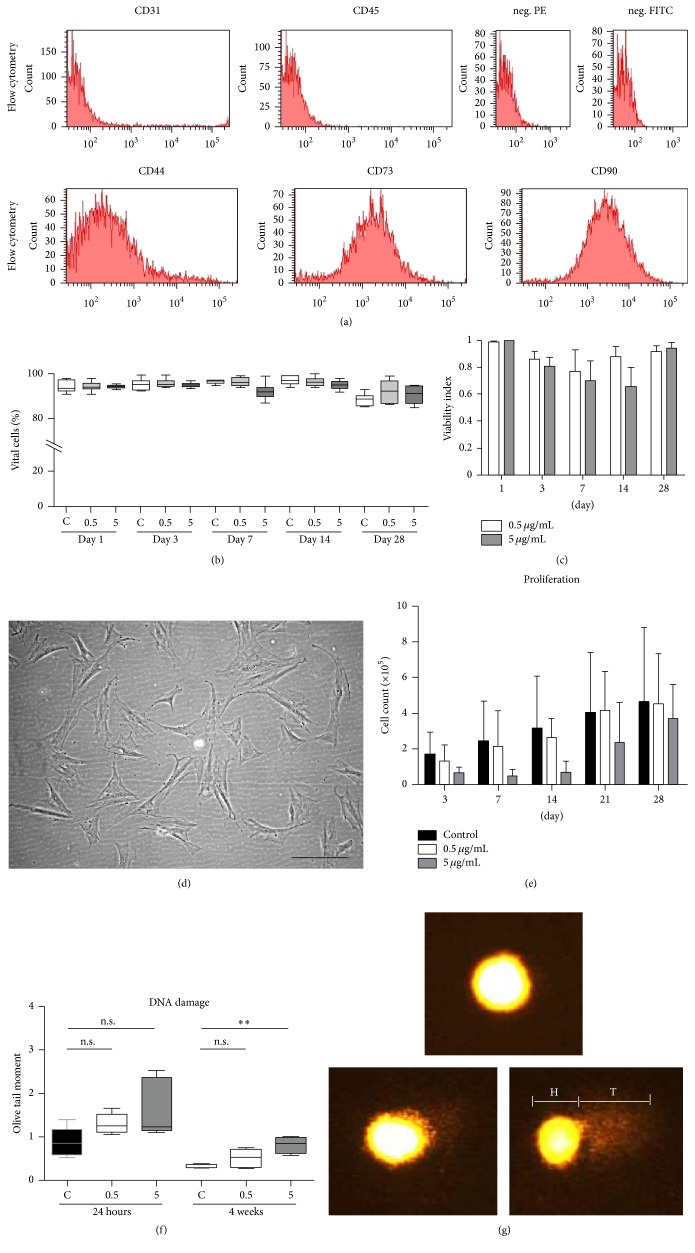
(a)* Histogram of rat ASCs flow cytometry* with antibodies against CD cell surface markers: the intensity of the fluorescence is shown on the *x*-axis and the cell counts are shown on the *y*-axis. The negative control (neg.) for PE- and FITC-conjugated antibodies (unstained cells) are shown in the upper right. An ASC-specific configuration with CD44, CD73, and CD90 being positive and CD31 and CD45 being negative was observed. (b)* FDA-PI vitality staining*: the percentage of vital cells is presented at day 1, 3, 7, 14, and 28 for control ASCs (“C”) and ASCs labeled with 0.5 *μ*g/mL (“0.5”) and 5 *μ*g/mL (“5”) H33342. Data is presented as the average of five experiments (error bars: SD). Normal vitality was observed for unlabeled cells and both types of labeled cells. Cells labeled with 5 *μ*g/mL Hoechst showed slightly decreased vitality after 7 and 14 days. (c)* MTT assay*: the viability index is shown for both labeled groups until day 28. Data is presented as the average of five experiments (error bars: SD). (d)* Phase contrast microscopy* of rat ASCs used in this study. Scale bar represents 200 *μ*m. (e)* Cell counts* at day 3, 7, 14, 21, and 28 for labeled (0.5 *μ*g/mL and 5 *μ*g/mL H33342) and unlabeled ASCs. Data is presented as the average of five experiments (error bar: SD). (f and g)* Comet assay*: (f) the olive tail moment (OTM) as a measure of DNA damage is shown 24 hours and 4 weeks after labeling with 0.5 *μ*g/mL (“0.5”) H33342 and 5 *μ*g/mL (“5”) and for unlabeled ASCs (“C”). Data is presented as the average of five experiments (error bar: SD); ns is not statistically significant, ^*∗∗*^ *p* < 0.01. Fluorescence images (g) of the comet assay of unlabeled (upper picture) and labeled ASCs (0.5 *μ*g/mL lower left picture; 5 *μ*g/mL lower right picture). The head of the comet (“H”, lower right picture) represents the undamaged DNA. A tail (“T”, lower right picture) is formed by damaged DNA and can be seen in particular after labeling with 5 *μ*g/mL H33342, but not in unlabeled cells. Please notice that the lower right image depicts an over average tail value for illustration purposes.

The ASC-specific cell surface marker configuration was confirmed by flow cytometry prior to the experimental application of all cells used throughout the experiments. The cells were negative for CD31 and CD45 and positive for CD44, CD73, and CD90 (Figure 1(a)) and had a characteristic morphological appearance (Figure 1(d)). A stable viability above 90% was observed for unlabeled cells and both types of labeled cells with the FDA/PI viability staining (Figure 1(b)). After 28 days, the fraction of vital cells tended to drop in all groups. However, nearly 90% of the cells were vital at all time points. At day 7 and day 14, the viability of the ASC labeled with 5 *μ*g/mL Hoechst was slightly decreased compared to the other two groups. However, the differences were not statistically significant. Besides that, the viability of H33342 stained ASCs was evaluated by the MTT assay that provides an indirect measure of the number of proliferating cells (Figure 1(c)). The viability index (ratio of extinction values of labeled and unlabeled cells) of both labeled groups slightly decreased compared to the controls at day 3 and day 7. At day 14, the viability index of the ASC labeled with 0.5 *μ*g/mL was close to 0.9, whereas the viability index of the ASC labeled with 5 *μ*g/mL further decreased to 0.65. However, this difference did not reach the level of statistical significance. After 28 days, the cytotoxic effect of the dye disappeared almost completely. Cell labeling with 0.5 *μ*g/mL H33342 did not interfere with proliferation as indicated by comparison with the unlabeled controls (Figure 1(e)). Neither of the proliferation rates differed significantly. The proliferation rate of cells labeled with 5 *μ*g/mL, however, was markedly lower in the beginning, and the cell counts were nearly unchanged during the first 14 days. Thereafter, the proliferation rate enhanced noticeably and the cell counts increased with the same slope compared to the control group.

### 3.2. DNA Damage

The comet assay was performed 24 hours and 4 weeks after labeling. After 24 hours, the tail length (TL) was significantly increased in cells labeled with 5 *μ*g/mL in comparison to the unlabeled cells. The tail length of unlabeled cells averaged 46.99 *μ*m (SD 3.36 *μ*m), whereas the TL of labeled cells were 48.63 *μ*m (SD 3.99 *μ*m) and 52.28 *μ*m (SD 3.66 *μ*m) after labeling with 0.5 *μ*g/mL and 5 *μ*g/mL H33342, respectively. The olive tail moment (OTM) values were not significantly increased 24 hours and 4 weeks after labeling with 0.5 *μ*g/mL H33342 (Figure 1(f)). However, cells labeled with 5 *μ*g/mL showed a noticeable increase in OTM values after 24 hours and a significant increase in OTM values after 4 weeks compared to the unlabeled controls, which implies relevant DNA damage Figure 1(f). The difference between ASCs labeled with 0.5 *μ*g/mL and unlabeled cells was not statistically significant at 4 weeks.

### 3.3. Multilineage Differentiation Potential

**Figure 2:**
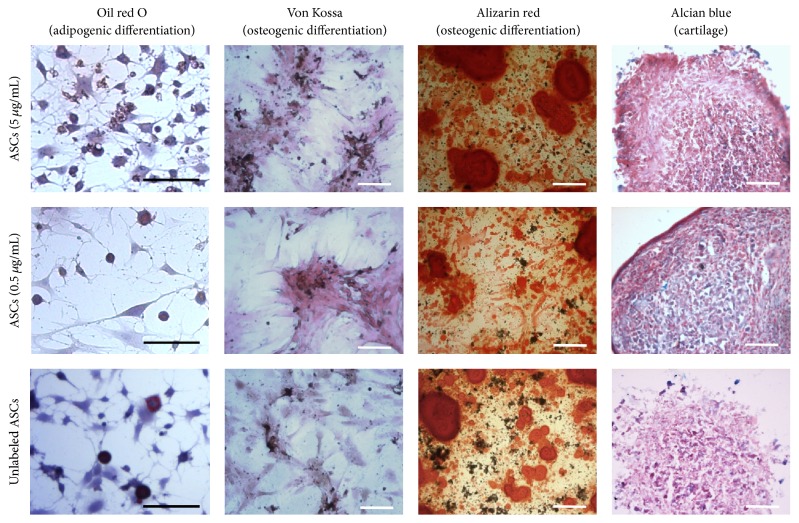
*Multilineage differentiation potential* of unlabeled ASCs and labeled ASCs. The differentiation was induced 24 hours after labeling. The first and second rows show ASCs labeled with 5 *μ*g/mL and 0.5 *μ*g/mL H33342, respectively, after the differentiation procedures. In the last row, unlabeled ASCs after differentiation are shown. First column: oil red O staining after adipogenic differentiation. Second and third column: von Kossa staining (black nodules detecting phosphates) and alizarin red staining (detecting red calcium deposits) after osteogenic differentiation. Last column: alcian blue staining detects glycosaminoglycans (GAGs) of the extracellular matrix after chondrogenic differentiation. Scale bar represents 100 *μ*m.

The multilineage differentiation potential was shown for H33342 labeled and unlabeled ASCs 24 hours (Figure 2) and 2 weeks after cell labeling, respectively. After adipogenic differentiation and oil red O staining in both labeled and unlabeled cells, intracellular lipid vacuoles were detectable. Osteogenic differentiation was detected after von Kossa and alizarin red staining. Black nodules after von Kossa staining (indicating phosphate) and calcium deposits in alizarin red staining were clearly visible in all groups in conjunction indicating a similar osteogenic differentiation potential. Glycosaminoglycans (GAGs) of the extracellular matrix were detected after chondrogenic differentiation and alcian blue staining. In all groups, GAGs were present indicating a similar chondrogenic differentiation potential. Accordingly, labeled and unlabeled cells exhibited a regular adipogenic, osteogenic, and chondrogenic differentiation potential.

### 3.4. Labeling Stability and Contamination

ASC labeled with 0.5 *μ*g/mL and 5 *μ*g/mL H33342 exhibited adequate fluorescence until day 28. At 28 days, 97.92% (SD 4.43%) of the ASC labeled with 0.5 *μ*g/mL and 99.28% (SD 1.21%) of the cells labeled with 5 *μ*g/mL were still clearly detectable. At 56 days after labeling, the percentage of adequately labeled ASCs significantly decreased to 43.28% (SD 16.95%) in the 0.5 *μ*g/mL group compared to 99.48% (SD 0.43%) after labeling with 5 *μ*g/mL.

**Figure 3:**
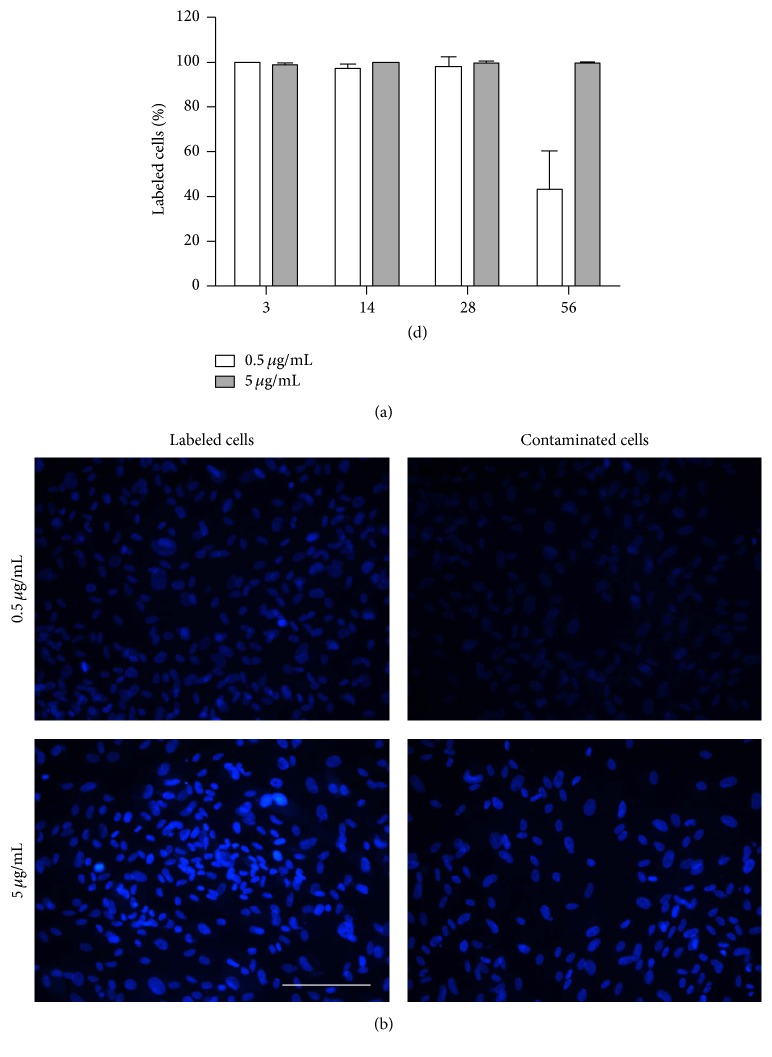
*Labeling stability* (percentage of labeled cells, a) of ASCs labeled with 0.5 *μ*g/mL or 5 *μ*g/mL H33342 over a period of 8 weeks.* Coculture of labeled and unlabeled ASCs on cover slips* (b): representative images with an exposure time of 200 ms after 8 days of coculture. In the left column, ASCs labeled with 0.5 *μ*g/mL (upper picture) and 5 *μ*g/mL H33342 (lower picture) are shown. The right column shows formerly unlabeled ASCs that were cocultured on the same cover slip. Scale bar represents 200 *μ*m.

In cocultures on cover slips with direct proximity between labeled and unlabeled cells, a distinct contamination of unlabeled cells with H33342 was observed. At all three time points (2, 4, or 8 days), almost all formerly unlabeled cells placed on the right side of the cover slip were labeled. Surprisingly, this observation applied for each of the three different washing procedures. However, there was a difference in the staining intensity depending on the H33342 concentration. Formerly, unlabeled cells cocultured with ASCs labeled by 0.5 *μ*g/mL H33342 were well visible using an exposure time of 200 ms and ASCs labeled with 5 *μ*g/mL were well visible using an exposure time of 100 ms. Compared to labeled cells, contaminated cells had a markedly weaker staining (Figure 3).

When labeled and unlabeled ASCs were cocultured in different chambers of the transwell plates, a contamination of unlabeled ASCs was only observed after staining with the higher concentration of H33342 (Table 1). Again, no differences were observed based on the washing procedure.

## 4. Discussion

Cell labeling is essential for various experimental approaches. For cell quantification, staining of the cell nucleus is reasonable. Additional desired outcomes of cell labeling are the determination of unaffected cell proliferation and viability, in the case of stem cells, an unaffected differentiation potential, and a low contamination of adjacent cells.

In this study, we investigated Hoechst 33342 for labeling the cell nucleus of ASCs. It represents a material, which is both easily applicable and inexpensive. We obtained ASCs from rats, representing one of the most commonly used mammalian animal models. Labeling ASCs with a low concentration of H33342 (0.5 *μ*g/mL) did not influence cell proliferation and differentiation behavior significantly. Higher concentrations of 5 *μ*g/mL H33342 diminished proliferation rates, whereas the differentiation was not affected. Two papers describe cytotoxic effects and reduced proliferation rates after using the same material for concentrations of 10.7 *μ*M (6 *μ*g/mL) in fibroblasts and 5 *μ*g/mL in lymphocytes, respectively [4, 10]. Loeffler et al. demonstrated that with lower concentrations of H33342 like 0.25 *μ*M (0.14 *μ*g/mL), this problem can be solved [4, 10]. Another group reported that incubation of rat bone marrow stromal cells (rBMSCs) with 1 *μ*g/mL for 12 hours did not cause cell apoptosis or necrosis [11]. Thus far, the influence of this staining material on the differentiation potential of ASCs has not been described in literature. However, Adamski et al. [12] reported on an inhibited differentiation of H33342 stained cells of the C2C12 and PC12 line that feature stem cell characteristics.

In FDA/PI viability staining, ASCs labeled with lower concentrations of H33342 exhibited a stable viability above 90%. In the MTT assay, a slight toxic effect was determined in ASCs labeled with 0.5 *μ*g/mL after one week and two weeks. This toxic effect on the cells continuously decreased at later points in time. Staining of ASCs with high concentrations of H33342 (5 *μ*g/mL) caused a noticeable reduction in viability. In the comet assay, no significant toxic effects were detected when ASCs were stained with 0.5 *μ*g/mL, but toxicity increased in ASCs labeled with the higher concentration of the material (5 *μ*g/mL). In the literature, several reports found that H33342 might induce cell necrosis and apoptosis [4, 5, 13]. The effect decreased using lower concentrations (0.14 *μ*g/mL or, resp., 1 *μ*g/mL) of the material [4, 11]. Parish did not observe any negative effects of H33342 on motility or migration patterns in their experiments with lymphocytes [5].

In this study, ASCs labeled with 0.5 *μ*g/mL and 5 *μ*g/mL H33342 showed a clearly detectable fluorescence up to 28 days. Almost 100% of the cells were stained. After 58 days, the percentage of labeled cells significantly decreased in the group of cells labeled with 0.5 *μ*g/mL. The percentage of labeled cells after labeling with 5 *μ*g/mL H33342 remained constant. This is consistent with reports in the literature. Parish and Brenan and Parish reported that H33342 is well retained in lymphocytes and showed a high fluorescence for several days [5, 14]. Other groups successfully used this material to label other types of cells for in vitro and in vivo studies. Xue et al. labeled amniotic epithelial cells with H33342 (5 mM). After 4 weeks, H33342 labeled cells were still present at the injection site [15]. Over a period of 2 weeks, Chen et al. investigated neural precursor cells derived from the olfactory bulb, which were labeled with 10 *μ*g/mL of H33342. They were applied to cochlear nucleus and could be traced for 2 weeks [16].

Contamination of H33342 on adjacent cells was examined in two different settings in this study. In experiments with cocultures of labeled and unlabeled cells grown in direct proximity on one cover slip, the majority of the unlabeled cells were distinctly stained. Washing steps had only a minor influence on the contamination. In the second setting, cells were cocultivated in transwell systems without direct cell contact. With the low concentration of 0.5 *μ*g/mL H33342, no redistribution was observed, whereas higher concentrations of the material caused contamination in formerly unlabeled cells. In this study, different washing procedures had no relevant impact. This indicates that contamination is caused by H33342 released from labeled cells. Some research groups reported that H33342 elutes from the labeled cells and leads to staining of adjacent cells [4, 13]. Mohorko et al. reported that rBMSCs labeled with 1 *μ*g/mL H33342 led to staining of 50% of adjacent cells after 6 hours and 90% after 1 week [11]. In contrast, various other studies found no extended elution effects [5, 14]. Xue et al. showed that broad washing procedures after labeling cells with H33342 reduced contamination [15]. Moreover, during the recent years, an active efflux of H33342 has been described in a so-called “side population” of many cell types [12, 17]. It is associated with cell immaturity and thus suggested as a marker of stemness and can also be found in ASCs [18].

In conclusion, Hoechst 33342 represents a cost-efficient, easily applicable material for staining the cell nucleus. Hoechst 33342 used at a low concentration of 0.5 *μ*g/mL does not seem to influence cell proliferation, vitality, toxicity, or the differentiation potential of ASCs in any relevant way. For coculture experiments or in vivo transplantation, the purpose and mode of application of H33342 should be carefully considered. In particular, higher labeling concentrations of H33342 appear to cause contamination of the surrounding cells. This is a drawback for the use of this material in particular for in vivo experiments.

## Figures and Tables

**Table 1:** Contamination of unlabeled ASCs by cocultured H33342 labeled ASCs (0.5 *μ*g/mL and 5 *μ*g/mL) in transwell plates after 2 days and 7 days. Two different washing procedures were performed. The occurrence of an undesired colabeling and the required exposition time for detection is indicated.

	2 days		7 days	
Washing procedure	Labeled and 2x rinsed	Labeled, rinsed, detached, centrifuged and washed, cultivated for 2 days, and 2x rinsed	Labeled and 2x rinsed	Labeled, rinsed, detached, centrifuged and washed, cultivated for 2 days, and 2x rinsed

H33342	Detection of colabeled cells			

0.5 *µ*g/mL	No	No	No	No
5 *µ*g/mL	Yes (200 ms)	Yes (200 ms)	Yes (200 ms)	Yes (200 ms)

## Abbreviations

ASC: Adipose-derived stem cells
CD: Cluster of differentiation
FCS: Fetal calf serum
FDA: Fluorescein diacetate
FITC: Fluorescein isothiocyanate
H33342: Hoechst 33342
MTT: 1-(4,5-Dimethylthiazol-2-yl)-3,5-diphenylformazan tetrazolium bromide
OTM: Olive tail moment
PBS: Phosphate buffered saline
PE: Phycoerythrin
PFA: Paraformaldehyde
PI: Propidium iodide
rBMSCs: Rat bone marrow stromal cells
TL: Tail length.
